# A Competitive Endogenous RNA Network Based on Differentially Expressed lncRNA in Lipopolysaccharide‐Induced Acute Lung Injury in Mice

**DOI:** 10.3389/fgene.2021.745715

**Published:** 2021-11-30

**Authors:** Xianxian Jia, Jinhui Huang, Bo Wu, Miao Yang, Wei Xu

**Affiliations:** Department of Pediatrics, Shengjing Hospital of China Medical University, Shenyang, China

**Keywords:** acute lung injury, high-throughput RNA sequencing, lncRNA, lncRNA-miRNA-mRNA network, competing endogenous RNA

## Abstract

Non-coding RNAs have remarkable roles in acute lung injury (ALI) initiation. Nevertheless, the significance of long non-coding RNAs (lncRNAs) in ALI is still unknown. Herein, we purposed to identify potential key genes in ALI and create a competitive endogenous RNA (ceRNA) modulatory network to uncover possible molecular mechanisms that affect lung injury. We generated a lipopolysaccharide-triggered ALI mouse model, whose lung tissue was subjected to RNA sequencing, and then we conducted bioinformatics analysis to select genes showing differential expression (DE) and to build a lncRNA-miRNA (microRNA)- mRNA (messenger RNA) modulatory network. Besides, GO along with KEGG assessments were conducted to identify major biological processes and pathways, respectively, involved in ALI. Then, RT-qPCR assay was employed to verify levels of major RNAs. A protein-protein interaction (PPI) network was created using the Search Tool for the Retrieval of Interacting Genes (STRING) database, and the hub genes were obtained with the Molecular Complex Detection plugin. Finally, a key ceRNA subnetwork was built from major genes and their docking sites. Overall, a total of 8,610 lncRNAs were identified in the normal and LPS groups. Based on the 308 DE lncRNAs [*p*-value < 0.05, |log2 (fold change) | > 1] and 3,357 DE mRNAs [*p*-value < 0.05, |log2 (fold change) | > 1], lncRNA-miRNA and miRNA-mRNA pairs were predicted using miRanda. The lncRNA-miRNA-mRNA network was created from 175 lncRNAs, 22 miRNAs, and 209 mRNAs in ALI. The RT-qPCR data keep in step with the RNA sequencing data. GO along with KEGG analyses illustrated that DE mRNAs in this network were mainly bound up with the inflammatory response, developmental process, cell differentiation, cell proliferation, apoptosis, and the NF-kappa B, PI3K-Akt, HIF-1, MAPK, Jak-STAT, and Notch signaling pathways. A PPI network on the basis of the 209 genes was established, and three hub genes (Nkx2-1, Tbx2, and Atf5) were obtained from the network. Additionally, a lncRNA-miRNA-hub gene subnetwork was built from 15 lncRNAs, 3 miRNAs, and 3 mRNAs. Herein, novel ideas are presented to expand our knowledge on the regulation mechanisms of lncRNA-related ceRNAs in the pathogenesis of ALI.

## Introduction

Acute lung injury (ALI) is one of the main causes of acute respiratory failure (Pan et al., 2018). ALI manifests through extreme inflammation, pro-inflammatory cytokine release, among other symptoms, leading to impaired gas exchange and edema (Han et al., 2018). The death rate of severe ALI was >40.0% (Pan et al., 2018). Approaches to ameliorate ventilator management have been widely used to provide supportive care for patients with ALI (Fan et al., 2005). Despite recent advances in comprehending the epidemiology along with the pathogenesis of the disease, clinical studies shows that some drug approaches are not effective in decreasing mortality rates in individuals with ALI (Raghavendran et al., 2008). Therefore, further research is urgently needed to unravel the molecular drivers of ALI development and to produce more effective treatments.

A growing body of research have suggested that next-generation sequencing technologies may unearth novel insights into gene expression changes in different biological processes and help better diagnose, treat and prognosis of several diseases (Hu et al., 2006; Rajendran et al., 2018). Moreover, long non-coding RNAs (lncRNA) have remarkably changed our comprehending of diseases (Mendell, 2016). LncRNA transcriptionally and post-transcriptionally regulates target gene expression but has no protein-coding role (Engreitz et al., 2016). Many lncRNAs are transcribed during development at specific times and in specific tissues and exhibit multiple splicing patterns. In addition, growing research evidence have documented that lncRNAs play significant biological roles modulating gene expression, cell proliferation, cell metabolism, differentiation along with apoptosis (Braconi et al., 2011; Rinn and Chang, 2012; Sana et al., 2012; Takahashi et al., 2014; Chang et al., 2016; Jiang et al., 2016). However, the full mechanism of lncRNA in LPS (lipopolysaccharide)-triggered ALI is still unclear.

In 2011, the concept of competing endogenous RNA (ceRNA), first raised by Sardina et al., became attracting (Salmena et al., 2011) and was subsequently verified by accumulating experimental evidence (Ebert et al., 2007; Lee et al., 2010; Poliseno et al., 2010; Wang et al., 2010; Jeyapalan et al., 2011). Evidence suggests that lncRNAs can serve as endogenous molecular sponges and indirectly regulate mRNA expression levels by competitively docking to miRNAs with miRNA response elements shared by the reverse complementary docking seed region. The leading of the ceRNA theory has showed a new mechanism for co-action between RNAs, which has expanded our comprehension of the function of lncRNAs (Tay et al., 2014). The complicated cross talk of the ceRNA network has been demonstrated in numerous diseases (Zhou et al., 2016; Kristensen et al., 2018), including respiratory diseases. For example, Nan et al. revealed that MALAT1, a ceRNA, sponged miR-194-5p and enhanced FOXP2 expression thereby mediated LPS-triggered apoptosis of human pulmonary alveolar epithelial cells (Nan et al., 2020). Besides, XIST knockout alleviates LPS-triggered apoptosis, as well as inflammatory damage in WI‐38 cells under acute pneumonia by targeting miR-370‐3p/TLR4 (Zhang et al., 2019). Similarly, Luo et al. also revealed that lncRNAs and mRNAs expression profiles were altered in alveolar macrophages (AM) of ALI rats at an early stage, which suggested that lncRNA4344 sponged miR-138-5p to accelerate pyroptosis in inflammatory responses of LPS-stimulated acute lung injury *via* targeting NLRP3 (Luo et al., 2021). Nevertheless, the mechanism of lncRNA and ceRNA pairs participated in lung injury remains to be illustrated. Furthermore, ceRNA raises a new viewpoint to explain the role of yet undefined lncRNAs in LPS-triggered ALI. Herein, on the basis of the results of our high-throughput RNA sequencing (RNA-seq) experiments, we conducted an all-around analysis and created a ceRNA network with the purpose of elucidating a modulatory mechanism of lncRNAs in ALI and screening important genes in the ALI-related ceRNA network with a narrower range and higher precision. In order to understand whether differentially expressed (DE) lncRNAs have the function of ceRNAs in ALI, we selected their miRNA target genes and sponge miRNAs to create lncRNA-miRNA-mRNA network. Besides, GO along with KEGG assessments were employed for the determination of DE mRNA in the ceRNA network to explore the major functional cascades of ALI. Furthermore, a PPI network was established, from which hub genes were selected. In order to better comprehend the pathogenesis of ALI, we also established the regulation module of lncRNA-miRNA-hub gene subnetwork, which provides a new perspective for the potential regulation mechanisms of lncRNA in ALI.

## Materials and Methods

### ALI Animal Model

All animal experiments were consented by the Experimental Animal Ethics Committee of China Medical University (2017PS086K). Male BALB/c mice aged 6–8 weeks (weight 18–22 g) were bought from Changsheng Biotechnology Company (Benxi, China). The ALI mouse model was formed via intratracheal inoculation of LPS, as described in a prior study (Liu et al., 2013). In summary, mice were anesthetized with 20% urethane (0.15 g/kg), exposed to the trachea, and intratracheal injection of *E. coli* O55:B5 (L-2880, Sigma, United States; 5 mg/kg; Mice in ALI group and control group were given *E. coli* O55:B5 (L-2880, Sigma, United States; 5 mg/kg, 100 mg LPS dispersed in 100 ml of 0.9% normal saline) and 50 μL 0.9% normal saline in trachea, respectively. Mice were raised upright, rotated left and right, and their skin was sutured for feeding. 24 h after injection, mice were killed under anesthesia and lung tissues were excised and fixed (in 4% PFA) and then H&E staining performed or stored at −80°C for follow-up experiments.

### H&E Staining

The left lung was fixed with 4% PFA, paraffin-embedded and sliced (3 μm). Tissue sections were dewaxed in xylene for 20 min, and then rehydrated (in 100, 95, 85, and 70% alcohol) for 10 min, followed by H&E staining. The specimen was stained in hematoxylin for 2 min and then rinsed in flowing water for 15 min. Eosin staining of the sections was done for 1 min, followed by dehydration in 70, 95, and 100% alcohol, made of xylene transparent and sealed in a neutral resin. The pathological changes were observed under microscope (Nikon, Japan).

### RNA Isolation and Qualification

Total RNA was collected from lung tissues employing TRIzol reagent (Invitrogen, Life Technologies, United States), per the manufacturer manual, and RNA quantification was performed by Novogene Experimental Department. Concisely, RNA degradation along with contamination were examined with 1% agarose gels. After that, a NanoPhotometer® spectrophotometer (Implen, CA, United States) was employed to check RNA quality. Afterwards, RNA quantity was gauged with the Qubit® RNA Assay Kit on a Qubit® 2.0 Fluorometer (Life Technologies, CA, United States), and RNA integrity was estimated with the RNA 6000 Nano Assay Kit of a 2100 Bioanalyzer system (Agilent Technologies, CA, United States).

### RNA-Seq

Total RNA from the ALI (*n* = 3) and control (*n* = 3) groups was extracted and quality controlled. Index of the reference genome was established by bowtie2 v2.2.8 and paired-end clean reads were aligned to the reference genome based on HISAT2 (Langmead and Salzberg, 2012)v2.0.4. HISAT2 was run with “--rna-strandness RF,” other parameters were set as default. NGS and subsequent bioinformatics analysis of high-quality clean data were obtained using Novogene Bioinformatics Technology (Beijing, China). RNA-seq was based on an Illumina HiSeq 4000 platform, and 150 bp paired-end reads were produced, basing on Illumina’s protocol.

### Expression Analysis

Cuffdiff (V.2.1.1) was applied to compute fragments per kilobase of exon per million fragments mapped (FPKMs) of both lncRNAs and coding genes in each sample (Trapnell et al., 2010). FPKMs were computed according to the length of the fragments, as well as reads count mapped to the fragment. The FPKM of transcripts in every gene group was added up to calculate the gene FPKM.

### Differential Expression Analysis

For differential expression assessment of lncRNAs and mRNAs, we adopted the Ballgown software (Frazee et al., 2015), which supplies statistical routines for establishing differential expression in transcript or gene expression data employing a model in the light of the negative binomial distribution (Frazee et al., 2014). *p*-values were corrected with the Benjamini-Hochberg approach. *p* < 0.05 along with a |log2 (fold change) | > 1 were adopted as thresholds for remarkable differential expression.

### RT‐qPCR Validation of DE Transcripts

We attempted to verify the differential expression reported in the RNA-seq assay by qPCR. Top 5 up-regulated and down-regulated DE LncRNAs were selected for verification. Concisely, RNA was collected from lung tissues with TRIzol reagent (9108, Takara, Japan), as described by the manufacturer. Then, cDNA was generated from 1 µg total RNA with the PrimeScript RT Reagent Kit (RR047A, Takara) with gDNA Eraser for lncRNA and mRNA and 1 µg total RNA was reversed into cDNA with riboSCRIPT™ (c11027-2) for miRNA. RT-qPCR was cnducted using the TB Green PCR Core Kit (RR820A, Takara) on an ABI 7500 system (Thermo Fisher Scientific, Waltham, MA, United States). The following cycling conditions were used: 95°C for 30 s, followed by 40 cycles of 95°C for 5 s and 60°C for 34 s. All primer sequences are shown in Table 1. The 2^−ΔΔCt^ approach was employed to determine relative expression. qRT-PCR was conducted with 12 biological replicates. GAPDH mRNA and U6 small nuclear RNA (RNU6) were used as normalization standards.

**TABLE 1:** The specific description of the primer sequences.

Name	Forward primer	Reverse primer
AQP5	5′-CCG​TGT​GGC​TGT​GGT​CAA​AGG-3′	5′-TCG​ATG​GTC​TTC​TTC​CGC​TCC​TC-3′
SPC	5′-CTG​AGA​TGG​TCC​TTG​AGA​TGA​G-3′	5′-TCA​TGA​TGT​AGC​AGT​AGG​TTC​C-3′
LNC_005406	5′-CCG​ATG​GCT​GAC​TAA​CGC​TCT​TG-3′	5′-AGT​AGG​TGA​AGT​CTC​AGG​CAG​TAG​TG-3′
Crnde	5′-GGT​GGA​AGG​AGG​TGA​TTT​AGA​AGA​CAG-3′	5′-AGG​TGA​GGT​GAA​GGG​ATG​GAA​GG-3′
LNC_006646	5′-CGC​CTC​TGC​AAT​CAA​GCT​CTC​C-3′	5′-CAC​CAG​ACT​CGA​CCA​GCA​AGA​AC-3′
LNC_002227	5′-GTC​GAG​GAA​AAT​GGA​AAA​AGG​AGG​A-3′	5′-AAG​TCG​TCA​AGT​GGA​TGT​TTC​TCA-3′
LNC_006985	5′-ACC​CAG​TAG​TTG​CTC​CTC​TTC​TCG-3′	5′-AGC​TGA​CCA​GTT​TGC​CAA​TGA​GTG-3′
LNC_005443	5′-GAT​GCC​GTG​TGC​TCA​GAA​GGA​AG-3′	5′-GAA​CCC​TCC​CAG​CCC​TAT​CCA​C-3′
LNC_006842	5′-AAC​CCA​GAA​ATG​AAC​CCA​CAC​ACC-3′	5′-TCT​TCT​ACA​CGA​TAG​CAG​CCA​GTT​G-3′
RP23-451K23.3	5′-AGA​TGG​GTA​TCC​TCG​GGC​TTT​GG-3′	5′-CGT​TGT​TGC​TGA​GAC​TGG​GAA​GG-3′
LNC_006493	5′-CCA​GAA​AGG​TCA​GCA​CGC​AGT​C-3′	5′-AGC​AGG​TGG​CAG​GAG​AAG​GAA​G-3′
GAPDH	5′-AAA​TGG​TGA​AGG​TCG​GTG​TGA​AC-3′	5′-CAA​CAA​TCT​CCA​CTT​TGC​CAC​TG-3′

### ceRNA Network Analysis

To predict the inter-regulatory relationship among mRNAs, lncRNAs, and miRNAs, the miRanda (http://www.microrna.org/microrna/, Version 3.3a) software with parameters of “- sc 140 - en -10 - scale 4 - strict” was uitilized. Briefly, the potential miRNA response element was identified within the sequences of lncRNAs and mRNAs. Then, the miRanda software was used to forecast miRNA binding seed sequence sites, and overlapping of the same miRNA binding site on both lncRNAs [*p* < 0.05 and |log2 (fold change) | > 1] and mRNAs [*p* < 0.05 and |log2 (fold change) | > 1] constructed a lncRNA-miRNA-mRNA interaction network. The ceRNA regulatory network of lncRNA-miRNA-mRNA was visualized using Cytoscape software (Version 3.8.0).

### GO and KEGG Enrichment Analyses

To evaluate the role of genes in the ceRNA network, GO analysis of DE mRNAs in this network was done via the R goseq with correction for gene length bias (Young et al., 2010). GO terms with adjusted *p*-value < 0.05 were regarded as remarkably enriched by DE genes. KEGG (http://www.genome.jp/kegg/) is a data resource for elucidating the high-level roles, as well as utilities of biological systems (Kanehisa et al., 2008; Young et al., 2010), consisting of the cell, organism, and ecosystem, from molecular level data, in particular large-scale molecular data sets produced by genome sequencing along with other high-throughput experimental technologies. We adopted the KOBAS software to check the statistical enrichment of DEmRNAs of the ceRNA network in KEGG pathways (Mao et al., 2005), and a corrected *p*-value < 0.05 was used as the truncation criterion.

### Creation of the PPI Network and Confirm of Hub Genes

The Search Tool for the Retrieval of Interacting Genes (STRING, http://www.string‐db.org/) was applied to find the PPI network between mRNAs in the ceRNA modulatory network, providing all-around interactions between proteins and genes. Cytoscape (3.8.0) was employed to view the PPI network. The Molecular Complex Detection (MCODE) app of Cytoscape was used to acquire the hub genes and the parameters of MCODE were consider as node score cutoff = 0.2, degree cutoff = 2, max. depth = 100 and k-core = 2. The genes with the highest MCODE-score were set as hub genes (Bandettini et al., 2012).

### Reestablish of the Crucial ceRNA Subnetwork

Every triple communication in the above ceRNA network was re-screen to create a new subnetwork. Genes were screened out based on the top five most remarkable DE lncRNAs and confirmation of the hub genes. The respective miRNA predictions were made and forecasted their binding sites. The crucial ceRNA subnetwork was reestablish. We verified the expression levels of 6 lncRNAs, 3 miRNAs, and 3 mRNAs of the ceRNA sub network. All primer sequences are shown in Supplementary Table S3.

### Statistical Analysis

All experiments were conducted in triplicate. The statistical R package was employed for data analysis. Data with normal distribution are given as the mean ± standard deviation. The statistical significance of differences between groups was determined by the Student’s t-test (unpaired). The statistical analysis was done using GraphPad Prism 8.0 software, and *p* <0.05 signified statistical significance.

## Results

### ALI in Mice

**FIGURE 1:**
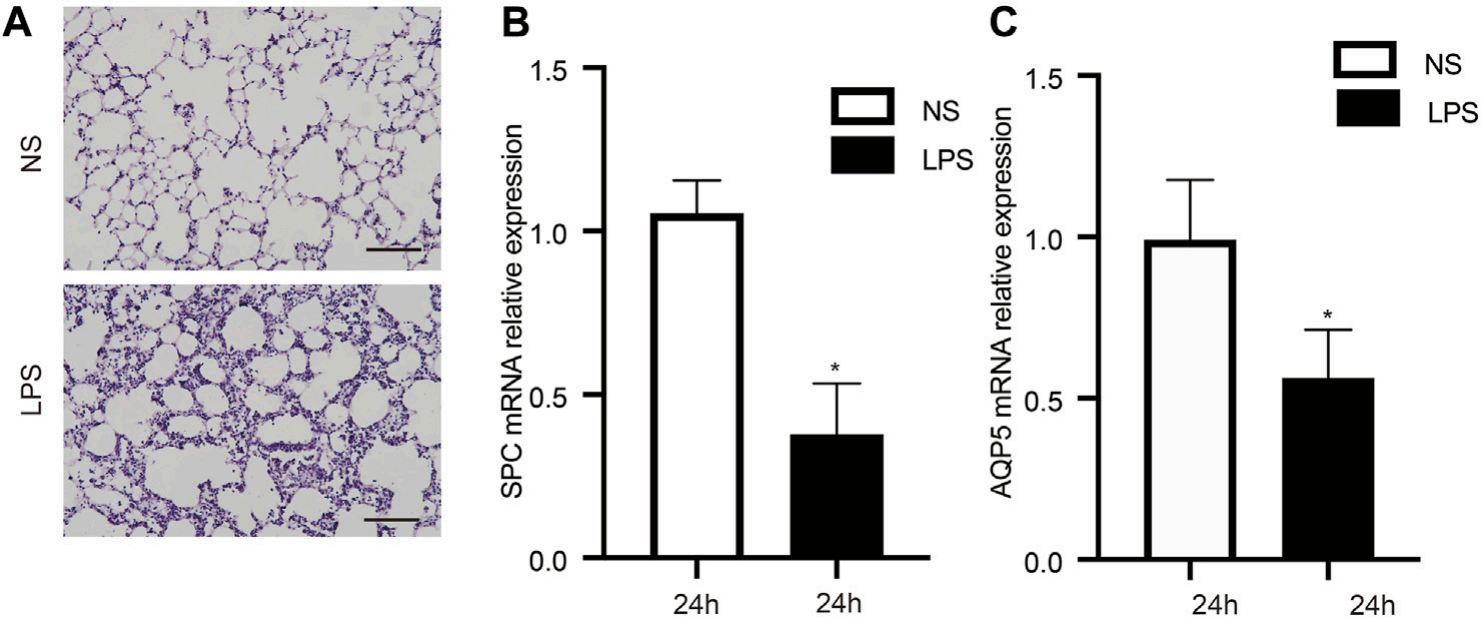
Characteristics of animals in the LPS-triggered and control groups. **(A)** Pathological observations in lung tissue stained with H&E. **(B, C)** LPS decreased the mRNA levels of SPC and AQP5. *n* = 12/group; *p < 0.05; **p < 0.01. LPS, lipopolysaccharide; NS, no lipopolysaccharide; SPC, surfactant protein C; AQP5, aquaporin 5; Scale bar: 40 µm.

The ALI model was triggered by LPS, and we observed that mice in this group exhibited shortness of breath and decreased autonomic activity, compared with the control group. Furthermore, H&E staining of tissues from the ALI group showed lung interstitial thickening and alveolar structure disorder (Figure 1A). Surfactant protein C (SPC) and water channel protein Aquaporin 5 (AQP-5) are two markers of alveolar epithelial type II cells (AECIIs) and alveolar epithelial type I cells (AECIs), respectively (Nielsen et al., 1997; Akella and Deshpande, 2013). The change of their expression level can indirectly reflect the injury of epithelial cells. Therefore, we observed remarkably decreased SPC (Figure 1B) and AQP5 mRNA (Figure 1C) measures in lung tissue from the ALI group, indicating AECIs and AECIIs damage.

### Characterization of ALI-Triggered lncRNAs and mRNAs in Lung Tissue

**FIGURE 2:**
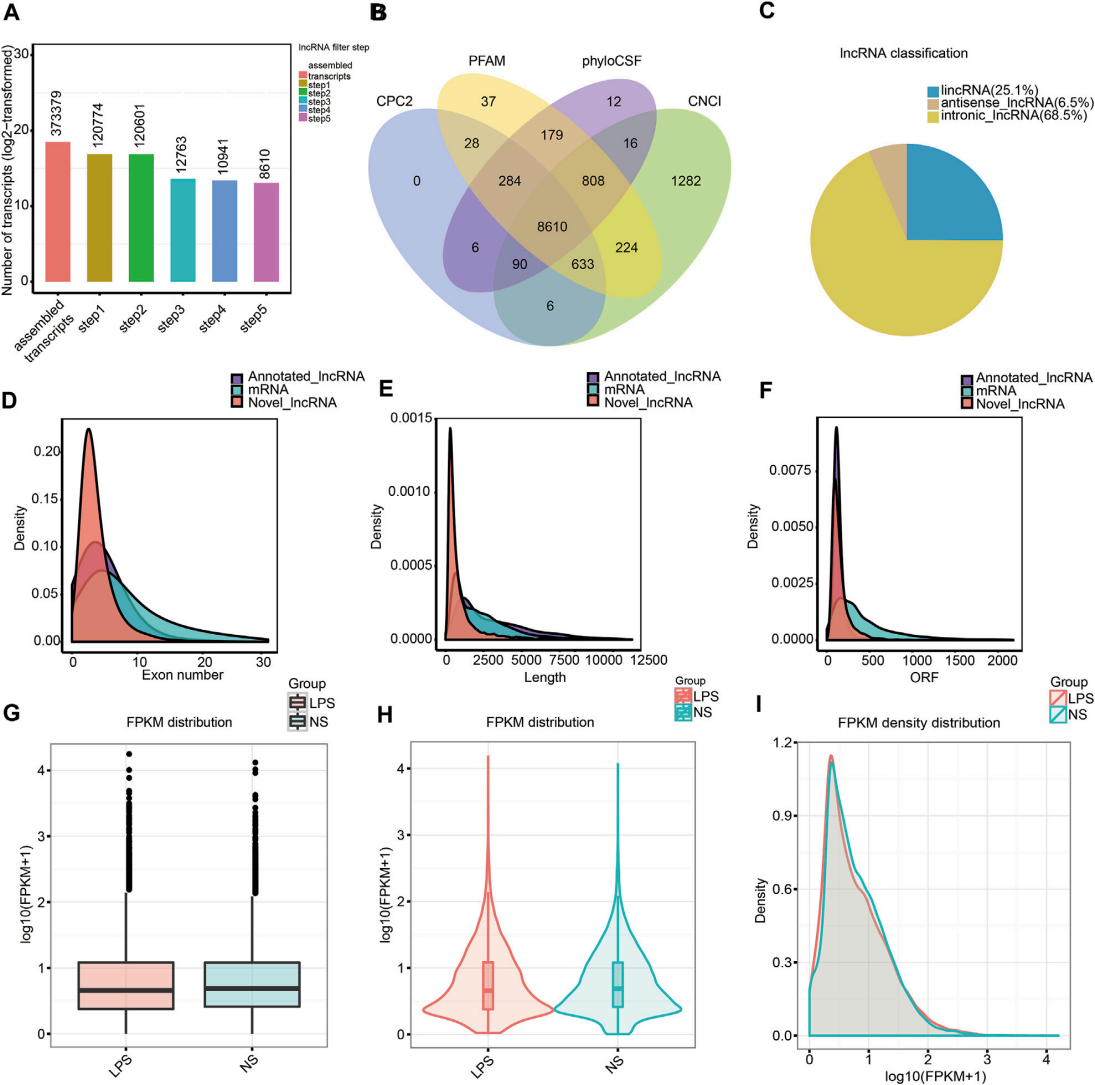
Characterizing lncRNAs along with mRNAs in mouse lung tissue. **(A)** Screening of new lncRNA in five steps. **(B)** Coding underlying analysis using CPC2, CNCI, PFAM, and phyloCSF. The sequences concurrently shared by the above four tools were chosen as candidate lncRNAs. **(C)** Classification of the uncovered lncRNAs. **(D–F)** Density distribution scheme illustrating **(D)** tthe expression characteristics of exon number, **(E)** length, and **(F)** ORF of annotated lncRNAs, new lncRNAs, as well as mRNAs in mouse lung tissue. **(G–I)** Boxplot **(G)**, violin plot **(H)**, and density distribution plot **(I)** revealing the expression characteristics of mouse lung tissue from the ALI group, as well as the control group. LPS, lipopolysaccharide; FPKM, fragments per kilobase of exon per million fragments mapped; NS, no lipopolysaccharide.

We assessed RNA-seq data from six mouse lung samples and obtained 93,446,460–107,777,762 and 90,677,668–103,975,066 raw and clean data, respectively. The classification of mapped reads in each lung tissue sample is exhibited in Supplementary Figure S1. After comparing the SNP, insertion or deletion, and alternative splicing events between the ALI and control groups, we discovered that the insertion or deletion counts were lower in the ALI group relative with the control group, and no remarkable differences in SNP and alternative splicing events were observed (Supplementary Figure S2). To narrow the false-positive rate of identified lncRNAs from 373,379 assembled transcripts, we exploited a lncRNA filter to discard transcripts that did not possess all characteristics of lncRNAs (Figure 2A). The five main steps are as follows: exon number screening of transcripts, transcript length screening, transcription known annotation screening, transcription expression screening, and coding potential screening (Supplementary Table S6). Using this method, we confirmed 8,610 novel lncRNAs from an intersection of the result analysis of CPC2, CNCI, PFAM, and phyloCSF (Figure 2B and Supplementary Table S7), which consisted of 2,152 lincRNAs (25.1%), 560 anti-sense lncRNAs (6.5%), and 5,898 intronic lncRNAs (68.5%) (Figure 2C). Meanwhile, 2216 annotated lncRNAs were identified. After identifying lncRNAs, we used both novel and annotated lncRNAs in the entire analysis. In addition, we discovered that annotated along with novel lncRNAs had fewer exons, a smaller size, and fewer ORFs relative to mRNAs (Figures 2D–F). Noteworthy, no remarkable difference in the FPKM distribution between lung tissue from the ALI and control groups was found (Figures 2G–I). The distribution of lncRNAs and mRNA in chromosome is shown in Supplementary Figure S3.

### ALI-Triggered Differential Expression and Cluster Analysis of lncRNAs and mRNAs in Lung Tissue

**FIGURE 3:**
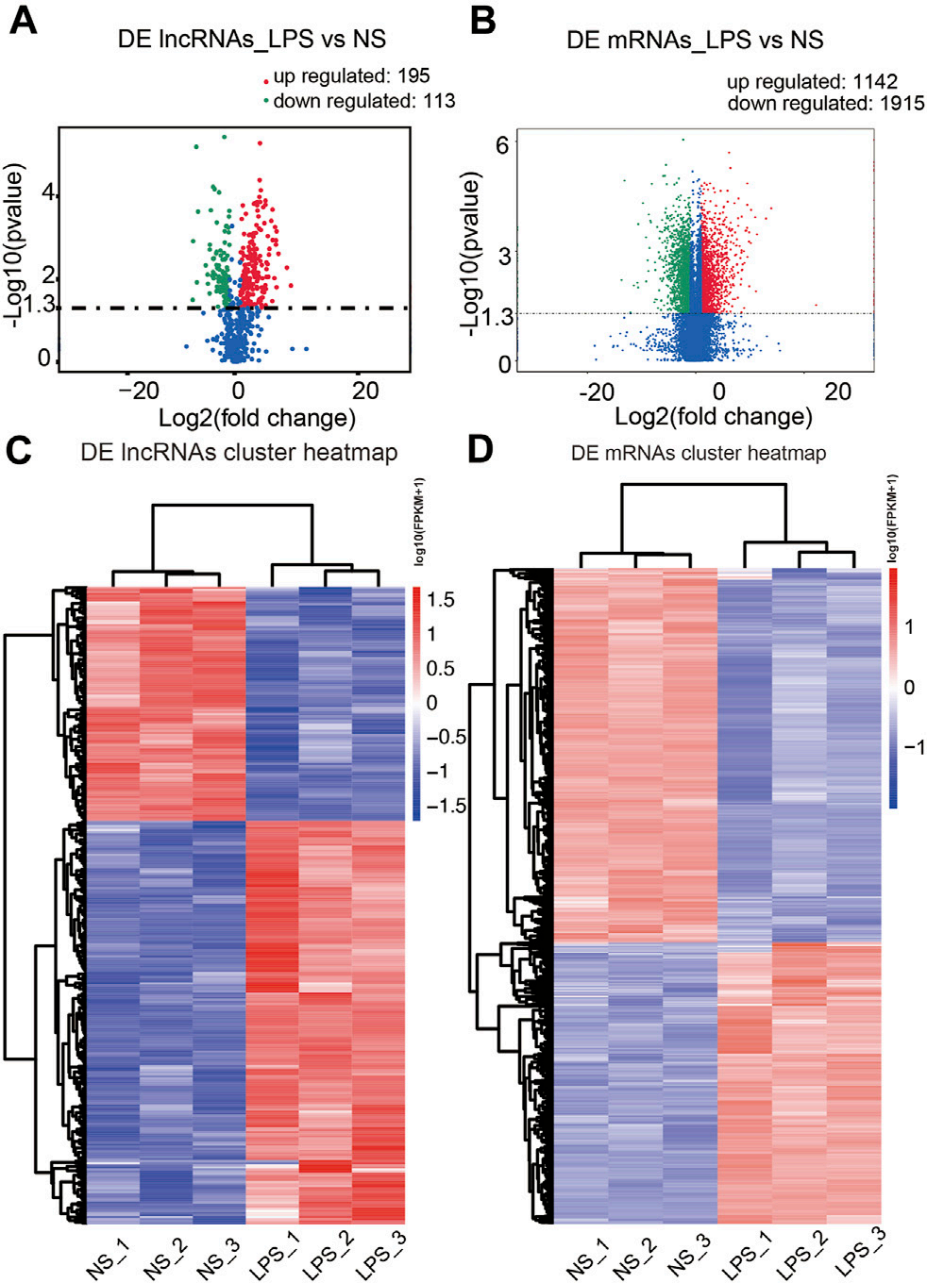
Comparison of the levels of lncRNAs and mRNAs in mouse lung tissues. **(A, B)** The volcano plots presenting the uncovered DE lncRNAs along with mRNAs in lung tissue from the ALI and control groups [*p* < 0.05 along with |log2 (fold change) | > 1.0]. Red designates upregulated genes, while green designates downregulated genes. **(C, D)** Hierarchical cluster analysis of the DE lncRNAs along with mRNAs in the ALI and control groups. Red, upregulated genes; blue, downregulated genes. LPS, lipopolysaccharide; NS, no lipopolysaccharide; DE, differentially expressed.

Based on transcripts per million data, we first uncovered 308 differential expression lncRNA transcripts in the ALI group [*p* < 0.05 along with |log2 (fold change) | > 1], including 195 upregulated and 113 downregulated transcripts, compared to the control group (Figure 3A). Cluster analysis of DE lncRNAs was conducted using a heatmap (Figure 3C). We used FPKM values to evaluate the mRNA contents. A total of 3,357 differential expression mRNA transcripts were uncovered in the ALI group, including 1,442 upregulated and 1,915 downregulated mRNAs [*p* < 0.05 along with |log2 (fold change) | > 1] (Figure 3B). The cluster analysis of DE mRNAs is shown in Figure 3D. The top 10 upregulated and top 10 downregulated lncRNAs in the ALI group are listed in Supplementary Table S1, and the top 20 upregulated along with top 20 downregulated mRNAs in the ALI group are showed in Supplementary Table S2. These data indicate that the expression level of lncRNAs and mRNAs was distinguishable and variable between both groups, and might contribute to the progression of ALI.

### qPCR Validation of the DE lncRNAs

**FIGURE 4:**
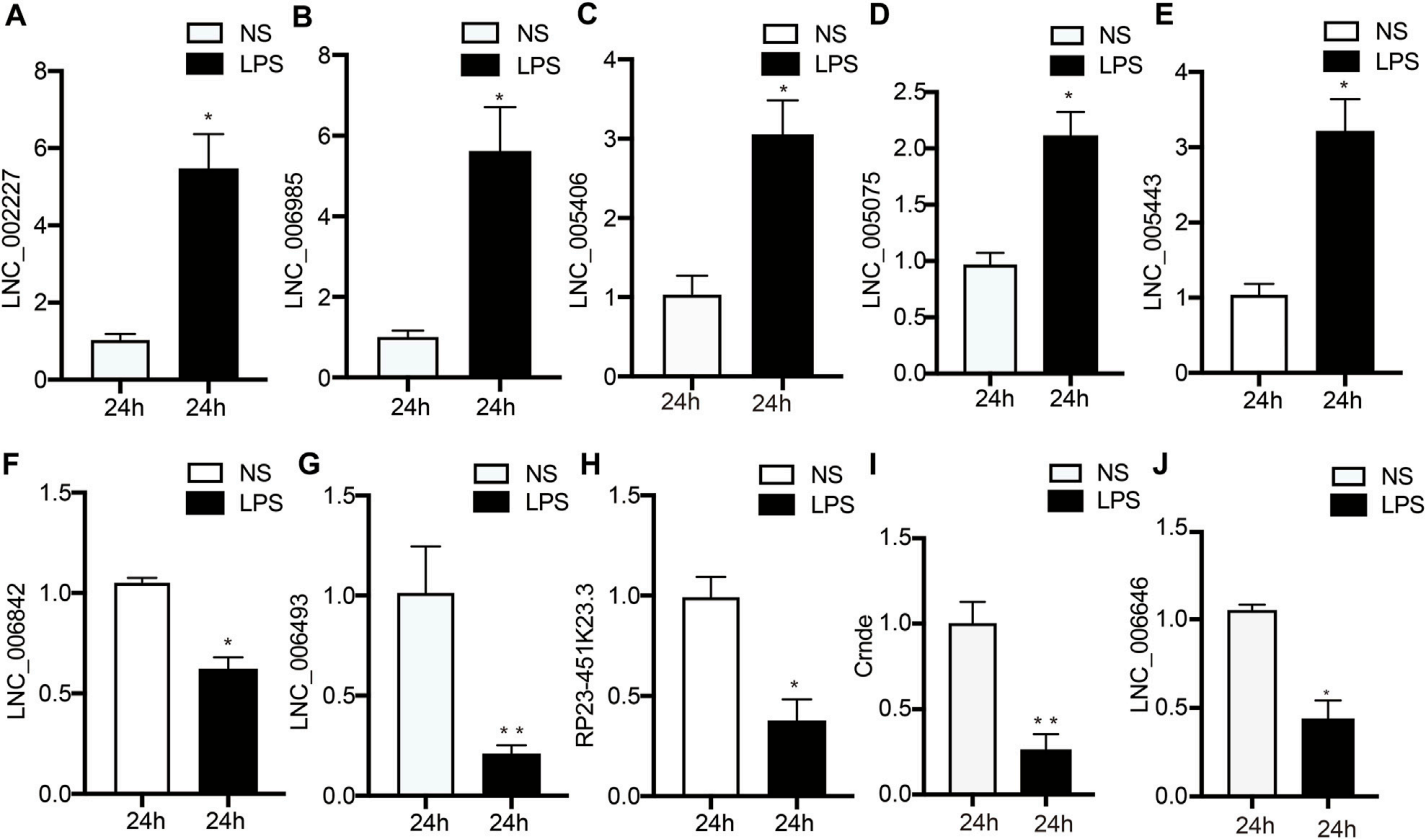
Validation of DE lncRNA expression using RT‐qPCR. **(A–I)** Changes in lncRNA expression were confirmed for DE lncRNAs in the LPS and NS groups by RT‐PCR. Data are given as the mean ± SEM (*n* = 12); *p < 0.05; **p < 0.01. LPS, lipopolysaccharide; NS, no lipopolysaccharide.

To verify the accuracy and credibility of the RNA-seq results and come up with a basis for further research, top 5 up-regulated and down-regulated DE lncRNAs were selected for RT-qPCR analysis. As shown in Figure 4, next-generation sequencing data were congruent with the RT-qPCR data regarding expression levels of the verified lncRNAs, indicating the high quality, as well as validity of RNA-seq data.

### Establishment of a lncRNA-miRNA-mRNA Modulatory Network

**FIGURE 5:**
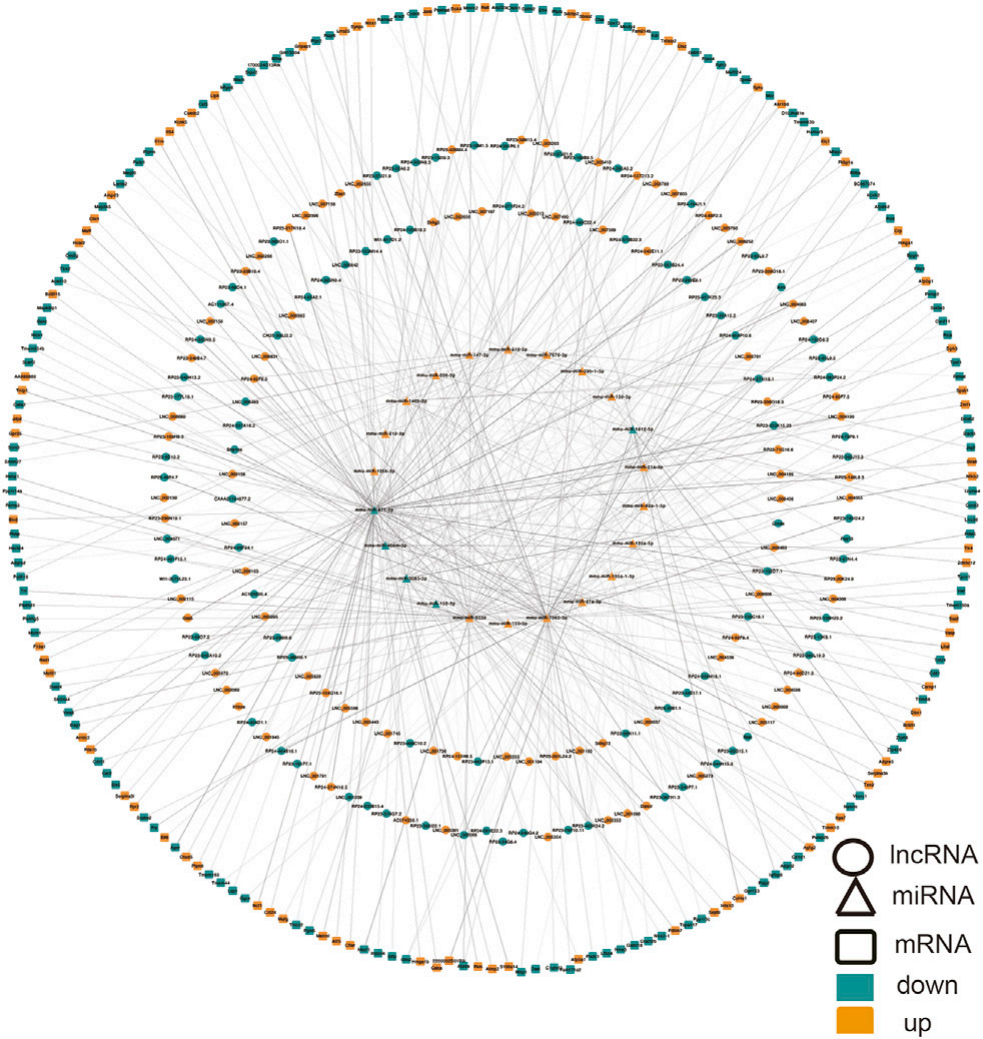
CeRNA network in ALI involving lncRNA-miRNA-mRNA. Ellipse, DElncRNA; triangle, DEmiRNA; rounded rectangle, DEmRNA. Red node, upregulation; blue node designates downregulation. miRNA, microRNA; ceRNA, competing endogenous RNA; DE, differentially expressed, lncRNA, long non-coding RNA.

We built ceRNA networks based on 308 DE lncRNAs and 3357 DE mRNAs. According to miRanda, among 175 DE lncRNAs were predicted to bind to 22 miRNAs and 209 mRNAs. The regulatory network was shown by Cytoscape software (Version 3.8.0) (Figure 5). Due to the presence of binding sites between lncRNAs and miRNAs, lncRNAs could indirectly modulate mRNA via competitively docking to miRNA, acting as a miRNA sponge. These RNA cross talks may provide a new perspective on the pathogenesis of ALI.

### Pathway and Functional Enrichment Analyses

**FIGURE 6:**
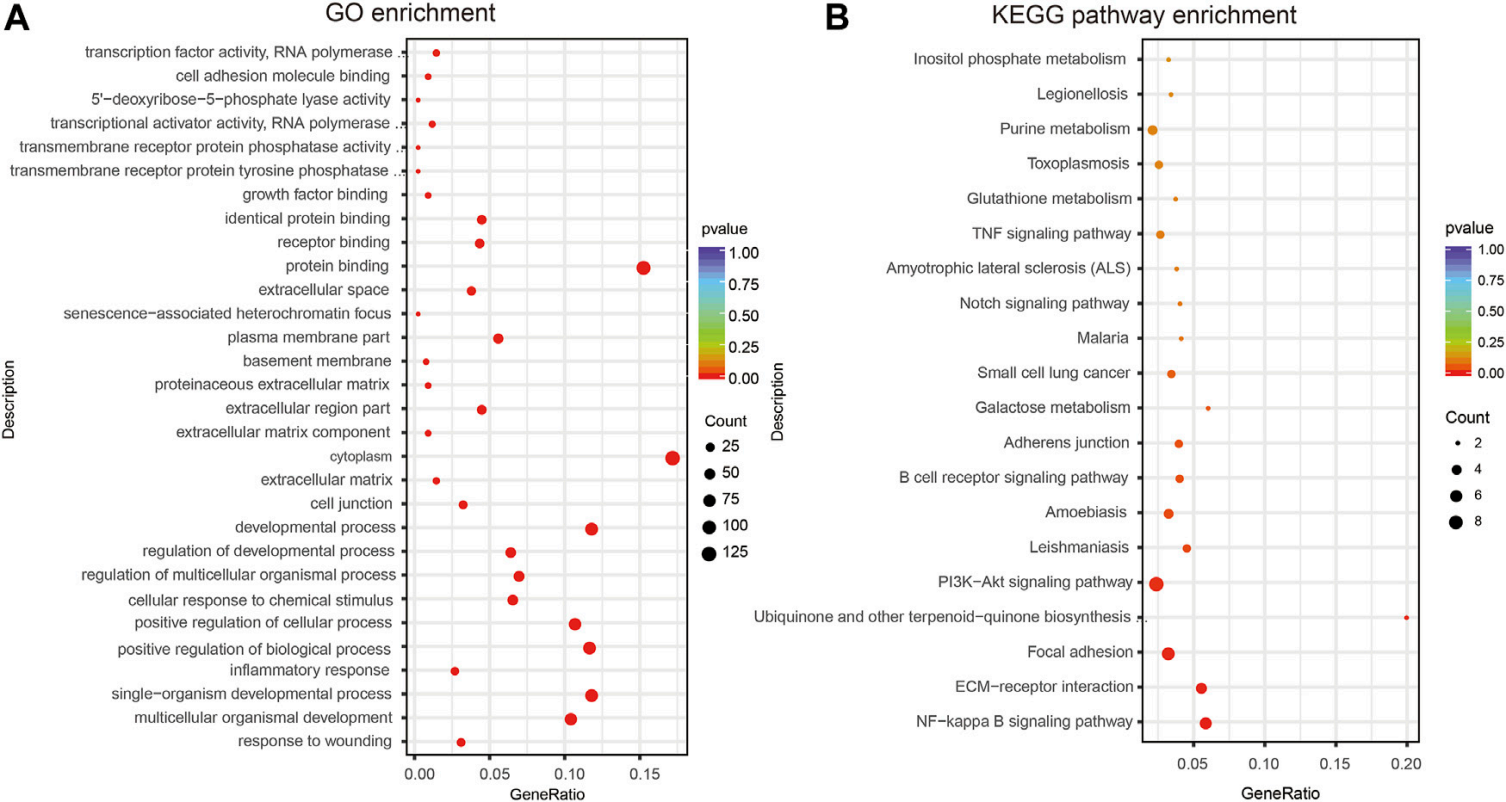
GO and KEGG analyses of DEmRNAs of the ceRNA network. **(A)** Ten most abundant GO terms (BP, CC, and MF) related to DEmRNAs participating in ceRNA network. **(B)** Twenty most enriched KEGG cascades related to DEmRNAs participating ceRNA network. BP, biological process; LPS, lipopolysaccharide; NS, no lipopolysaccharide; DE, differentially expressed; CC, cellular component.

The functions performed by lncRNA-related ceRNA networks are reflected in the associated mRNA genes. GO assessments were conducted on these genes, and 1,207 GO terms were discovered to be remarkably enriched (*p* < 0.05). The top 10 highly enriched GO terms of biological process (BP), cellular component (CC), and molecular function (MF) are shown in Figure 6A and Supplementary Table S4. GO data revealed that the most remarkably enriched terms were “inflammatory response,” “developmental process,” and “positive regulation of cellular process” in BP. Several other ALI-related terms were also observed, such as “cell differentiation,” “cell proliferation,” “cell death,” “MyD88-dependent toll-like receptor signaling pathway,” and “metabolic process.” Regarding CC, “cell junction,” “extracellular matrix,” and “cytoplasm” were obviously enriched terms, while “protein binding,” “receptor binding,” and “growth factor binding” were remarkably enriched in MF. KEGG cascade analysis was performed to establish the signaling cascades in which the related mRNAs participate. Ten remarkably enriched pathways were obtained (*p* < 0.05; Figure 6B and Supplementary Table S5). Among these, “NF-kappa B signaling pathway,” “PI3K-Akt signaling cascade,” “B cell receptor signaling pathway,” and “Galactose metabolism” are associated with the development of ALI. Additionally, other pathways, such as “Apoptosis,” “HIF-1 signaling pathway,” “MAPK signaling pathway,” “Jak-STAT signaling pathway,” “Notch signaling pathway,” “Rap1 signaling pathway,” “Ras signaling pathway,” “p53 signaling pathway,” “TGF-beta signaling pathway,” “TNF signaling pathway,” along with “FoxO signaling pathway,” were also seen to be enriched. Although these enrichment pathways were not statistically significant, several previous studies had reported significant changes of these signal pathways in the ALI. Therefore, we think that these pathways still need further explorations in the following research. All in all, the LncRNA-linked ceRNA network involves in the pathological process of ALI in different ways.

### PPI Network Creation and Hub Gene Analysis

**FIGURE 7:**
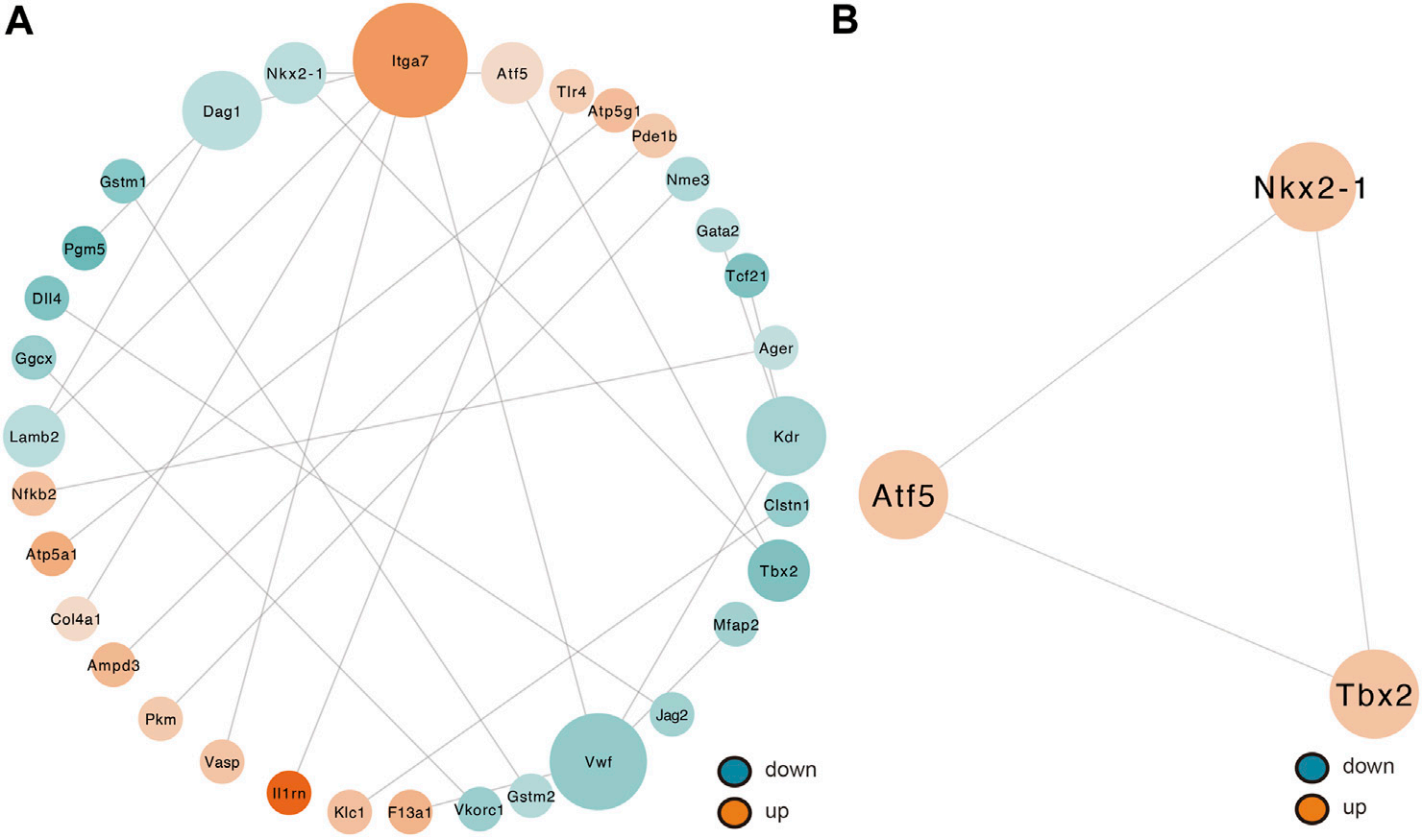
Determination of hub genes from the PPI network by the MCODE algorithm. **(A)** PPI networks of 209 target genes that play an indispensable role in ALI. This network makes up 33 nodes and 24 edges. The node color shifts from green to red gradually in ascending order, basing on the log2 (fold change) of genes. The node size shifts from small to large gradually in ascending order, basing on neighbored gene numbers per gene. **(B)** PPI network with the three hub genes isolated from **(A)**. This network makes up of three nodes and three edges. PPI, protein–protein interaction; MCODE, Molecular Complex Detection.

**FIGURE 8:**
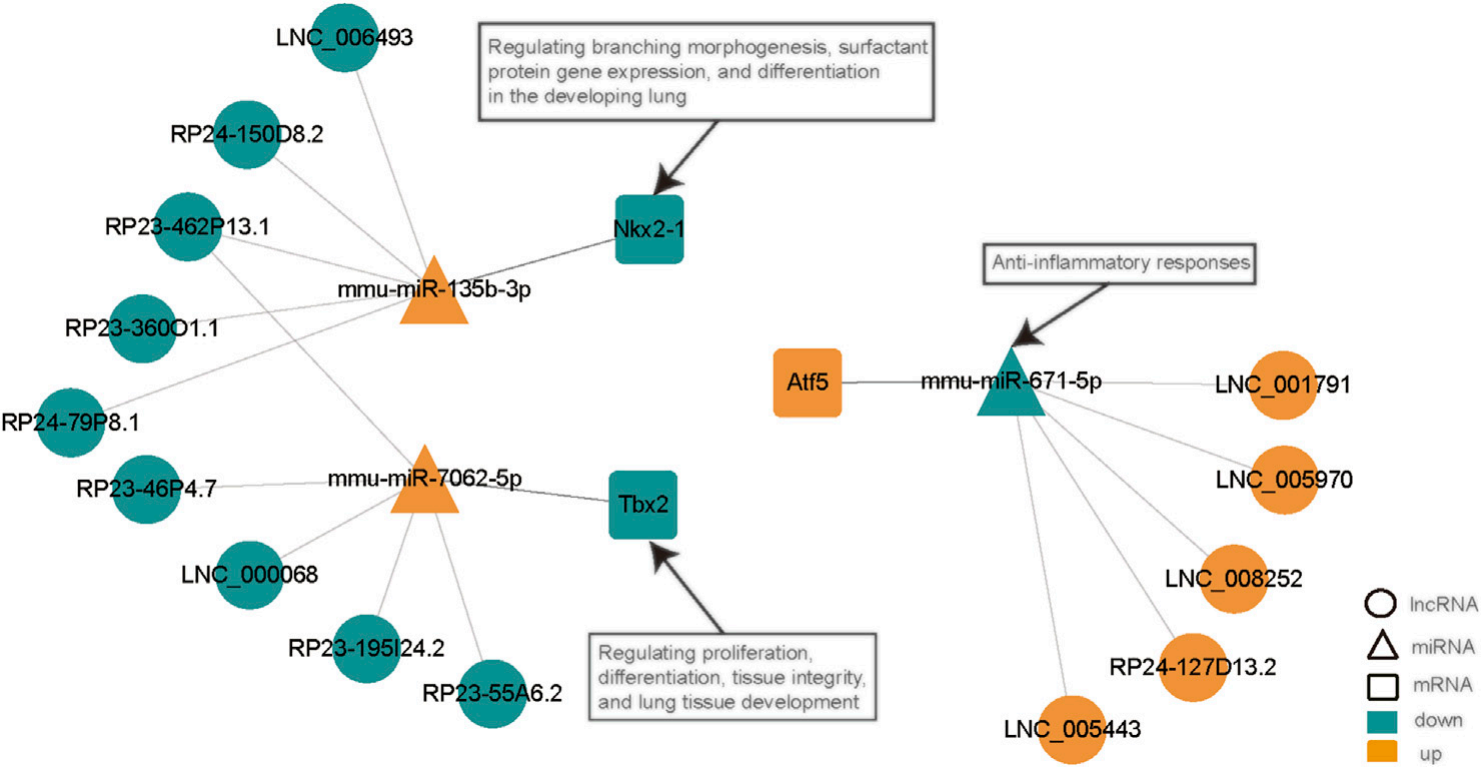
LncRNA-miRNA-hub gene network. This network, constituting 15 lncRNAs, 3 miRNAs, and 3 hub genes, was generated with Cytoscape (3.8.0). Ellipse, lncRNA; triangle, miRNA; rounded rectangle, mRNA. Red nodes, upregulation; dark green nodes, downregulation. LPS, lipopolysaccharide; ceRNA, competing endogenous RNA; NS, no lipopolysaccharide; lncRNA, long non-coding RNA; DE, differentially expressed; miRNA, microRNA.

By removing disconnected nodes, a PPI network was created, constituting 33 nodes and 24 edges, to observe the cross talks between the 209 mRNAs (Figure 7A). Given the importance of hub genes in a network, we used an MCODE method to screen hub genes from the PPI network. One subnetwork with three nodes and three edges was identified (k-core = 2; Figure 7B), which revealed the key role of the three genes (Tbx2, Nkx2-1, and Atf5) in ALI. The expression of Tbx2 and Nkx2-1 decreased, whereas that of Atf5 increased in ALI. Thereafter, a lncRNA-miRNA-hub gene network was created to describe the associations among the five most important DE lncRNAs, miRNAs and hub genes (Figure 8). Meanwhile, we verified the expression levels of 6 lncRNAs, 3 miRNAs, and 3 mRNAs of the ceRNA sub-network. As shown in Supplementary Figure S4, the expression level of the next-generation sequencing data was consistent with that of RT-qPCR data, which further demonstrated the quality and validity of RNA-seq data.

## Discussion

ALI and its aggressive form—acute respiratory distress syndrome—are remarkable factors of morbidity and mortality in patients with critical disease (Rubenfeld et al., 2005). Although the mechanism of lung injury varies according to its cause, epithelial and endothelial cell damage has been shown to be a critical factor in the onset and progress of ALI/acute respiratory distress syndrome (Tomashefski, 2000). LncRNAs remarkably influence many complicated human diseases, including lung injury (Li et al., 2018; Li et al., 2020). However, the accurate mechanism of lncRNAs in LPS-triggered ALI is not fully elucidated. Our study may unveil the lncRNA expression profile in LPS-triggered ALI and identify and analyze relevant lncRNAs and their connections using bioinformatics.

Unrecognized for a long time, lncRNA has undergone a significant breakthrough given the emergence of high-throughput sequencing and is now considered as a research hot point in the field of medicine. In this study, 8,610 lncRNA were selected in LPS-induced ALI by high-throughput sequencing. We summarized expression patterns and conducted a bioinformatics analysis of lncRNAs that may be participate in the pathogenesis of ALI. We identified 308 DE lncRNAs by screening [*p* < 0.05, |log2 (fold change) | > 1]. According to bioinformatics tools for target prediction, among 308 DE lncRNAs, 175 conformed to ceRNA network rules. A lncRNA-miRNA-mRNA ceRNA network was created. We also generated a model PPI network with DE mRNAs. In addition, we established a lncRNA-miRNA-hub gene subnetwork in the light of modulatory modules uncovered in the miRNA-lncRNA-mRNA network. Next, top 5 up-regulated and down-regulated DE lncRNAs were chosen for RNA-seq data verification using RT-qPCR. The results herein suggests that lncRNAs levels might take participate in the onset and progress of ALI.

To elucidate the biological role and possible mechanisms of lncRNAs in ALI pathogenesis, GO along with KEGG pathway analyses were conducted, which revealed a close relationship with: the inflammatory response; developmental process; positive modulation of the cellular process; cell proliferation, differentiation, migration, and death; MyD88-dependent toll-like receptor signaling cascade; and metabolic process. ALI disrupts the alveolar epithelium, leading to inflammation, an important feature of ALI pathology (Standiford et al., 1991; Jin and Dong, 2013). Moreover, the chronic induction of developmental cascades in aging is bound up with numerous lung pathologies (Königshoff et al., 2008; Selman et al., 2008; Chanda et al., 2016; Joannes et al., 2016). Most of these developmental cascades remain inactive in the adult, however are stimulated during injury repair, being considered an important factor in epithelial repair during lung injury (Selman et al., 2008). Yang et al. revealed that lipoxin A4 alleviates lung injury by stimulating epithelial cell proliferation (Yang et al., 2019). Sun et al. found that TAZ is a key factor in AECIIs to AECIs differentiation and, therefore, in maintaining alveolar integrity after injury (Sun et al., 2019). Cell migration, as well as growth are indispensable for a normal embryonic development, angiogenesis, immune system function, and repair of lung epithelial wounds after injury (Liu et al., 2019). Furthermore, multiple genes and signaling pathways, including the NF-kappa B (Gross et al., 2018; Wu et al., 2018), PI3K-Akt (Meng et al., 2018; Zhou et al., 2018), and B cell receptor (Han et al., 2014) signaling pathways and galactose metabolism (Bucior et al., 2013), have been repeatedly reported in ALI studies. Some other pathways, such as apoptosis and the HIF-1, MAPK, Jak-STAT, Notch, Rap1, Ras, p53, TGF-beta, TNF (Lai et al., 2019), and FoxO (Seiler et al., 2013) signaling cascades, have also been found to be enriched. Apoptosis is deemed as directly bound up with the severity of ALI, according to several studies focused on the development and manifestations of ALI (Ferrari and Andrade, 2015; Tian et al., 2017). For instance, remarkable apoptosis of the alveolar epithelial cells of type II has been illustrated to be the cause of impaired epithelial barrier function, as well as remodeling of certain mesenchymal cells in ALI (Bardales et al., 1996). McClendon et al. revealed that HIF-1a is stimulated in alveolar epithelial type II cells following lung injury and enhances proliferation along with migration during repair (McClendon et al., 2017). Pan et al. suggested that miR-124 alleviates the symptoms of ALI through dampening the induction of the MAPK signaling cascade *via* successive targeting of MAPK14 (Pan et al., 2019). In addition, Zhao et al. demonstrated the protective influence of repressing STAT3 activity in LPS-triggered ALI (Zhao et al., 2016; Jin et al., 2018). Another research illustrated that Notch signaling is stimulated in alveolar epithelial type II cells following alveolar injury and that DLK1-triggered Notch repression is necessary for complete AECIIs to AECIs transformation and alveolar repair (Finn et al., 2019).

Herein, all DE lncRNAs were found to contain miRNA response elements that sponge different miRNAs. The transcripts showing the greater differences in expression were screened out based on the top five most remarkable DE lncRNAs with the potential to form a lncRNA—miRNA-hub mRNA modulatory network. Among those lncRNAs, LNC_006493, RP24-150D8.2, RP23-462P13.1, RP23-360O1.1, and RP24-79P8.1 were found to be downregulated and predicted to bind to mmu-miR-135b-3p, which was upregulated in this study. Meanwhile, the downregulated RP23-46P4.7, LNC_000068, RP23-462P13.1, RP23-195I24.2, and RP23-55A6.2 were predicted to positively regulate the function of mmu-miR-7062-5p. In addition, LNC_005970, LNC_001791, LNC_008252, RP24-127D13.2, and LNC_005443 were found to be upregulated and predicted to bind mmu-miR-671-5p, which was downregulated in this study. miR-671-5p has been shown to be linked to anti-inflammatory responses. Lien et al. showed that inhibition of miR-671-5p in orbital fat-originated stem cells enhances the anti-inflammatory response in LPS-triggered ALI (Lien et al., 2014). Notably, miR-135b-3p and miR-7062-5p have not been reported thus far. Therefore, we speculate that these lncRNAs have the underlying function of particularly sponging miR-135b-3p, miR-7062-5p, or miR-671-5p to control the expression of subsequent target genes in ALI. Nonetheless, additional research is necessary to elucidate the roles of lncRNAs and the modulatory mechanisms of ceRNAs in ALI.

Regarding the DEmRNA-derived PPI network, the nodes with the higher scores—Nkx2-1, Tbx2, and Atf5—were screened as hub genes. A lncRNA-miRNA-hub gene network was then created to describe the associations among the top five most remarkable DE lncRNAs, miRNAs, and hub genes. Our results suggest that the downregulated LNC_006493, RP24-150D8.2, RP23-462P13.1, RP23-360O1.1, and RP24-79P8.1 may be particularly important for ALI, as they may function as ceRNAs for the upregulated mmu-miR-135b-3p, thus, leading to the downregulation of Nkx2-1. Moreover, the downregulated RP23-46P4.7, LNC_000068, RP23-462P13.1, RP23-195I24.2, and RP23-55A6.2 were predicted to positively regulate the function of mmu-miR-7062-5p, consequently downregulating Tbx2. Besides, we established that LNC_005970, LNC_001791, LNC_008252, RP24-127D13.2, and LNC_005443 may function as ceRNAs that block the repression influences of mmu-miR-671-5p on the expression of Atf5. NKX2.1 is a nuclear protein and member of the NKX2 family of homeodomain transcription factors. NKX2.1 plays an important role in the developing lung by regulating branching morphogenesis, surfactant protein gene expression, and differentiation (Keijzer et al., 2001; DeFelice et al., 2003; Jacob et al., 2017; Chen et al., 2018). In the lung, the expression of NKX2.1 is limited to alveolar epithelial type II cells (Lopez-Rodriguez et al., 2016). Abnormal changes in NKX2.1 expression is conducive to the onset of human diseases, for instance lung diseases. Reduced or absent expression of NKX2.1 is seen in areas of acute inflammation, atelectasis, as well as bronchopulmonary dysplasia, in the lungs of infants (Wert et al., 2002; Chen et al., 2012; Young et al., 2013; Lopez et al., 2015). On the contrary, NKX2.1 expression is escalated in regenerating lung regions. The role of NKX2.1 in lung diseases may be because of its potential to modulate SP-A, SP-B, and SP-C expressions (Yang et al., 2006; Islam and Mendelson, 2008; Zhou et al., 2008; Knabe et al., 2016). Liu et al. also revealed that increased NKX2.1 level may boost lung epithelial remodeling following injury by elevating cell migration along with proliferation (Liu et al., 2019). T-box (TBX) genes code for a family of evolutionarily conserved transcription factors involved in organogenesis, as well as development (Naiche et al., 2005; Hariri et al., 2012). TBX genes play important roles in proliferation, differentiation, as well as tissue integrity (Packham and Brook, 2003; Papaioannou, 2014). In mice, the TBX2 subfamily (TBX2/3/4/5) members are emerged in the developing lungs (lung buds along with trachea) (Gibson-Brown et al., 1998; Horton et al., 2008). TBX2 was revealed to be essential in maintaining normal cell proliferation in the lung mesenchyme of mice by depleting the gene (Lüdtke et al., 2013). Moreover, TBX2-deficient embryonic lungs showed decreased branching, whereas normal lung branching was mainly triggered by TBX2 along with TBX3 (Lüdtke et al., 2016). TBX2 content is reduced in differentiated fibroblasts and is necessary for the lung tissue development (Wojahn et al., 2019). Jiang et al. demonstrated that ATF4, but not ATF5, mediates the response of mitochondrial unfolded protein in alveolar epithelial cells in response to various mitochondrial stressors (Jiang et al., 2020). Furthermore, they discovered that the inducible ATF4 expression in mouse alveolar epithelial cells aggravates the mitochondrial unfolded protein response and inflammation in the lungs, induces body weight loss, and leads to death in response to bleomycin-triggered lung injury. Whether Nkx2-1, Tbx2, and Atf5 have a corresponding function in ALI is still unknown and requires further study.

The data of this study reveal promising critical genes, cascades, and ceRNA modulatory networks related to ALI, which may contribute to the development of targeted treatments. Nevertheless, there are some limitations to our study. Firstly, the large-scale lncRNA-miRNA-mRNA crosstalk network did not indicate whether these RNAs are co-expressed in the same tissues. Secondly, although the PCR results validated the credibility of the RNA-seq data, only three samples were employed for sequencing in each group; thus, the modulatory networks or mechanisms explored in this study were only according to bioinformatics predictions and there is a lack of practical experiments to validate these results. Hence, more all-around analyses and further thorough *in vitro* and *in vivo* studies using lncRNA silencing or overexpression are necessary. At present, our team is working on studying these complicated and potential mechanisms.

In summary, we screened DE lncRNAs, miRNAs, and mRNAs from RNA-seq data from mice with ALI to create lncRNA-associated ceRNA and PPI networks. The lncRNA-miRNA-hub genes modulatory subnetwork revealed critical genes and cascades that might take participate in ALI, providing a new idea for in-depth comprehension of the pathogenesis of this disease and supplying underlying regulation mechanisms for further research.

## Data Availability

The data presented in the study are deposited in the NCBI GEO repository, accession number GSE188492. https://www.ncbi.nlm.nih.gov/geo/query/acc.cgi?acc=GSE188492.

## Abbreviations

ALI, acute lung injury; AQP5, aquaporin 5; BP, biological process; CC, cellular component; ceRNA, competing endogenous RNA; DE, differentially expressed; FPKM, fragments per kilobase of exon per million fragments mapped; GO, gene ontology; KEGG, Kyoto Encyclopedia of Genes and Genomes; lncRNA, long non-coding RNA; LPS, lipopolysaccharide; MCODE, Molecular Complex Detection; MF, molecular function; mRNA, messenger RNA; miRNA, microRNA; PPI, protein–protein interaction; RNA-seq, RNA sequencing; SPC, surfactant protein C; TBX, T-box.
