# Supplementary figures and images for: A Competitive Endogenous RNA Network Based on Differentially Expressed lncRNA in Lipopolysaccharide‐Induced Acute Lung Injury in Mice
Source: Front Genet. 2021 Nov 30;12:745715. doi: 10.3389/fgene.2021.745715 (PMC8669720; doi:10.3389/fgene.2021.745715)

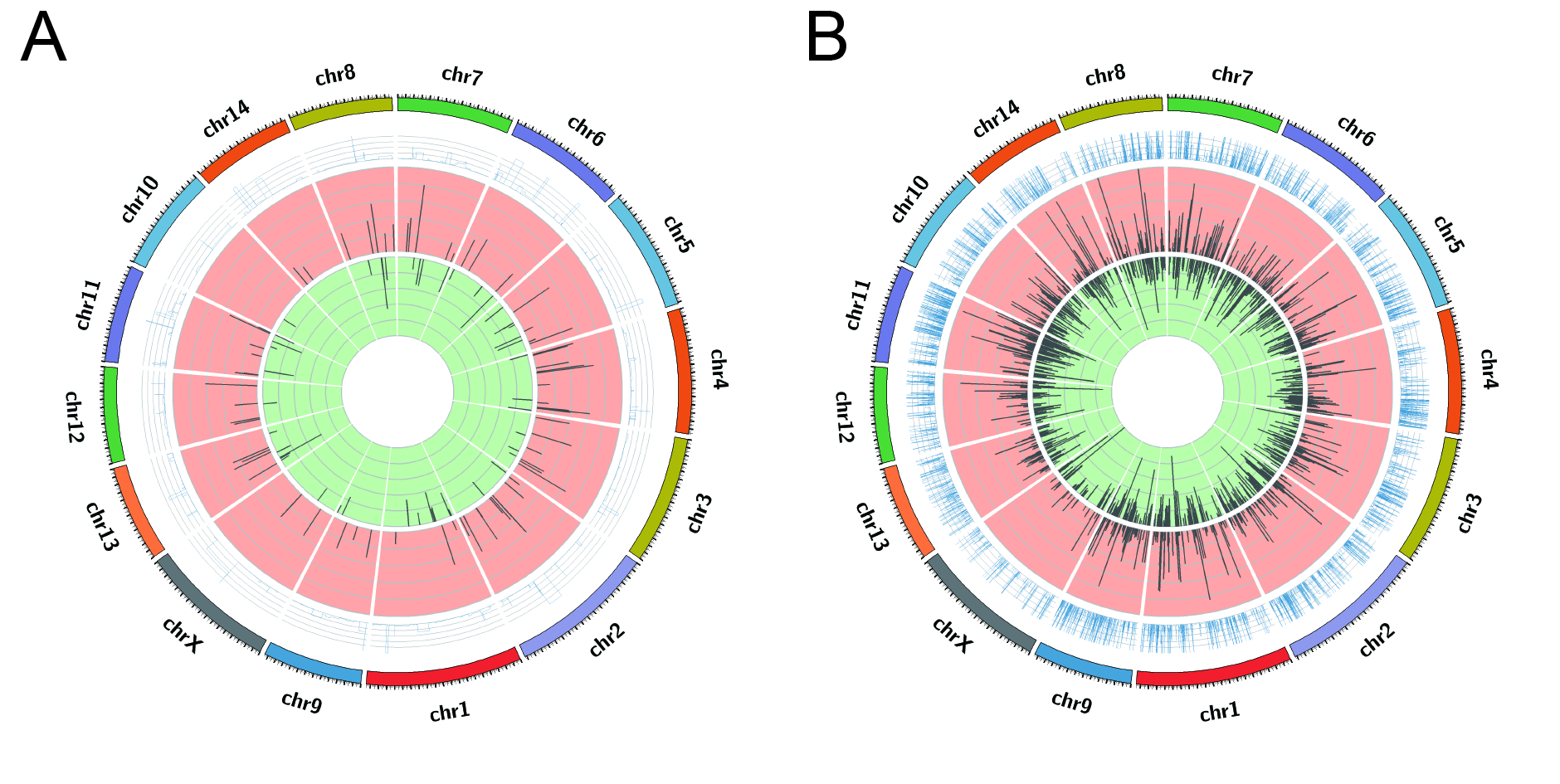

Supplement: Supplementary file 1 [file Image3.TIF]

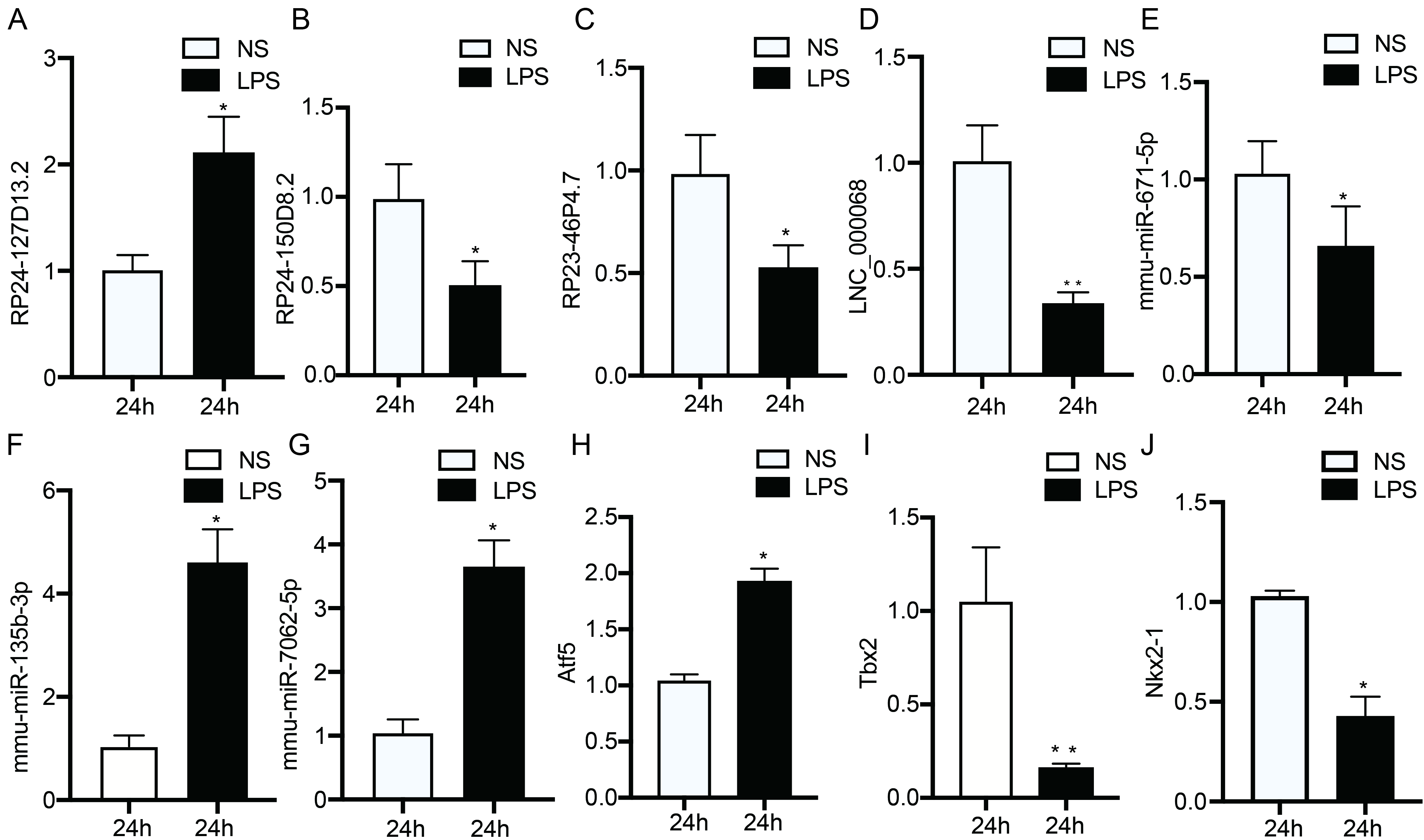

Supplement: Supplementary file 2 [file Image4.TIF]

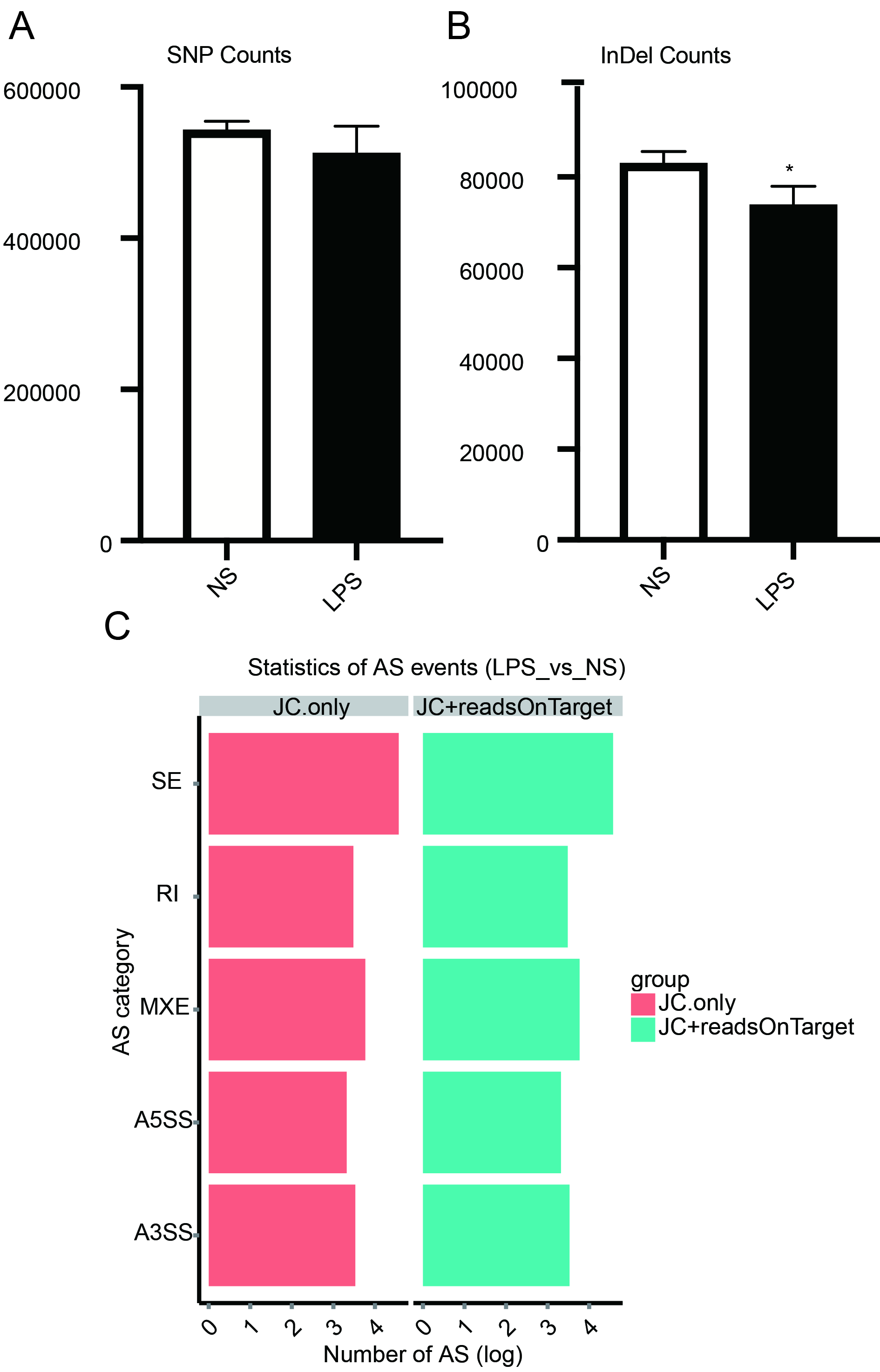

Supplement: Supplementary file 3 [file Image2.TIF]

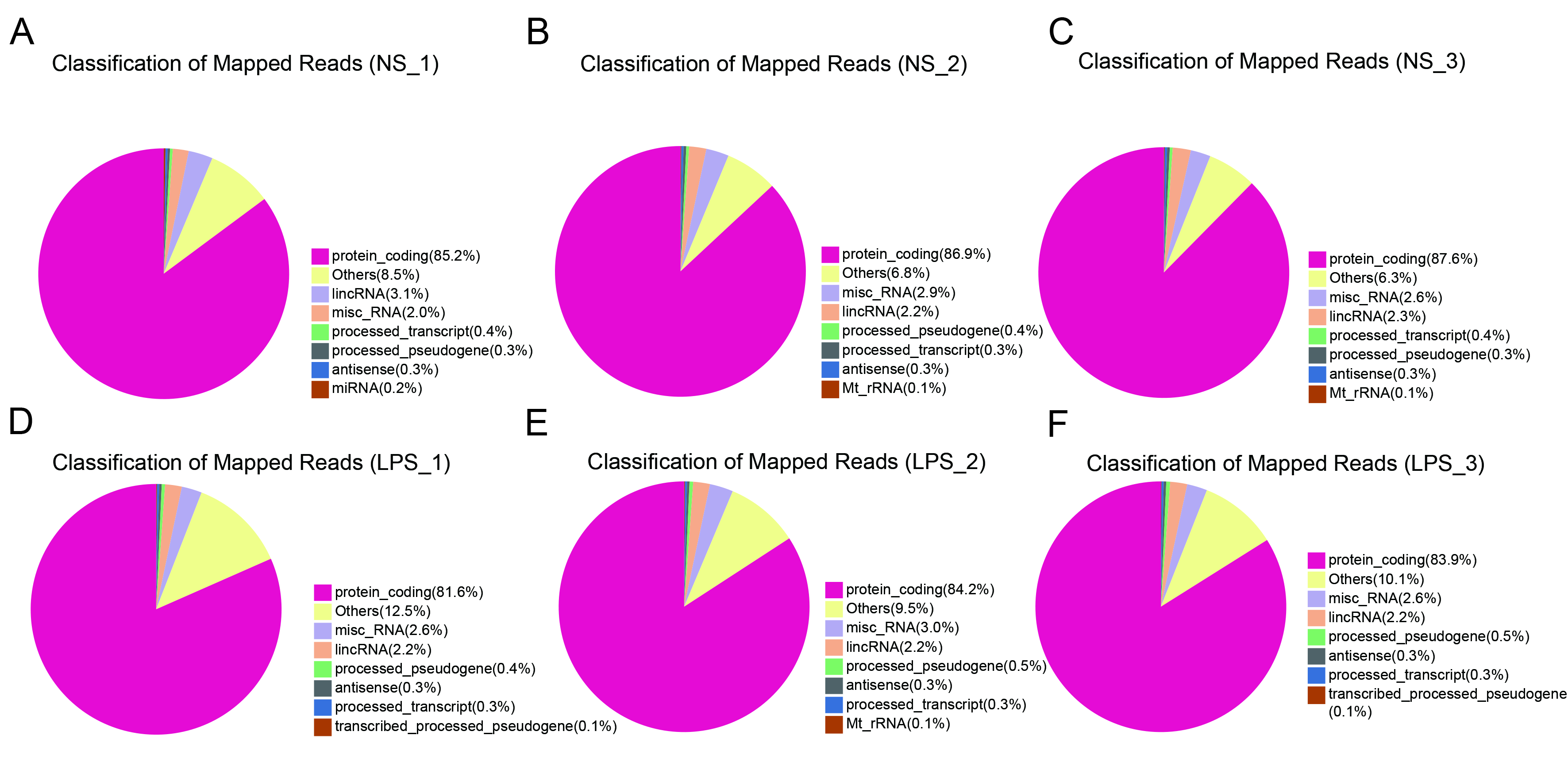

Supplement: Supplementary file 4 [file Image1.TIF]
