# Supplementary material for: A Competitive Endogenous RNA Network Based on Differentially Expressed lncRNA in Lipopolysaccharide‐Induced Acute Lung Injury in Mice
Source: Front Genet. 2021 Nov 30;12:745715. doi: 10.3389/fgene.2021.745715 (PMC8669720; doi:10.3389/fgene.2021.745715)
Supplement: Supplementary file 5 [file DataSheet1.docx]

Supplementary Material

## 1.Supplementary Figures and Tables

**
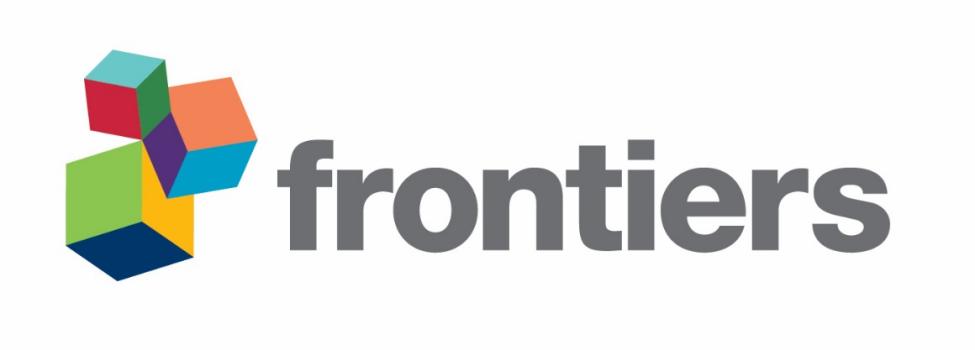
**

**1.1 Supplementary Figures**

**Supplementary Figure S1.** The classification of mapped reads in mouse lung tissue.

**Supplementary Figure S2.** The single nucleotide polymorphisms (SNP) counts (A), insertion-deletion (InDel) counts (B), and alternatively spliced (AS) events (C) in lung tissue. RI: retained intron; JC, junction counts; A5SS: alternative 5' splice site; SE: skipped exon; A3SS: Alternative 3' splice site; MXE: mutually exclusive exon.

**Supplementary Figure S3.** Distribution of lncRNAs (A) and mRNA (B) in chromosome.

**Supplementary Figure S4. Validation of DE gene expression using RT‐qPCR. (A–I)** Changes in gene expression were confirmed in the LPS and NS groups by RT‐PCR. Data are given as the mean ± SEM (n = 12); *P < .05; **P < .01. LPS, lipopolysaccharide; NS, no lipopolysaccharide.

**1.2 Supplementary Tables**

**Supplementary Table1.** The specific description of the top 10 up-regulated and 10 down-regulated lncRNAs.

| transcript_id | lncRNA | log2.foldchange. | pvalue | Regulation |
| --- | --- | --- | --- | --- |
| LNC_002227 | LNC_002227 | 10.45960573 | 0.014592203 | up |
| LNC_006985 | LNC_006985 | 9.704299535 | 0.005402786 | up |
| LNC_005406 | LNC_005406 | 8.124491242 | 0.002489112 | up |
| LNC_005075 | LNC_005075 | 7.773803101 | 0.000715789 | up |
| LNC_005443 | LNC_005443 | 7.768434955 | 0.016073424 | up |
| ENSMUST00000173070.7 | RP23-349B4.7 | 7.664159556 | 0.001182662 | up |
| LNC_003940 | LNC_003940 | 7.654047053 | 0.001503526 | up |
| LNC_005838 | LNC_005838 | 7.496430571 | 0.000562096 | up |
| LNC_001870 | LNC_001870 | 7.369360613 | 0.009623708 | up |
| LNC_004353 | LNC_004353 | 7.052311062 | 0.00116157 | up |
| ENSMUST00000154798.7 | RP23-57F11.3 | -4.703246013 | 0.001962345 | down |
| ENSMUST00000124439.1 | RP23-46P4.7 | -4.733359287 | 0.022552441 | down |
| ENSMUST00000206679.1 | RP23-68N11.1 | -5.042414746 | 0.013146485 | down |
| ENSMUST00000145030.1 | CH36-125B10.3 | -5.114714068 | 0.007902554 | down |
| ENSMUST00000229383.1 | RP24-544N15.3 | -5.460109552 | 0.004737869 | down |
| LNC_006842 | LNC_006842 | -6.894789839 | 0.0002389 | down |
| LNC_006493 | LNC_006493 | -7.108857576 | 0.012161864 | down |
| ENSMUST00000220673.1 | RP23-451K23.3 | -7.218337996 | 6.53E-06 | down |
| ENSMUST00000179222.1 | Crnde | -7.810289659 | 0.001228891 | down |
| LNC_006646 | LNC_006646 | -7.888650179 | 0.032207604 | down |

**Supplementary Table2.** The specific description of the top 20 up-regulated and 20 down-regulated mRNAs.

| mRNA | log2FoldChange | P-val | Regulation |
| --- | --- | --- | --- |
| Mff | 22.22634979 | 0.029716941 | up |
| Adcy7 | 13.93971906 | 6.74E-05 | up |
| Gm15056 | 12.97067481 | 0.000126849 | up |
| Lcp1 | 11.37303439 | 5.72E-06 | up |
| Csf3 | 11.30321766 | 0.000345783 | up |
| Ikbke | 11.12004535 | 0.012495979 | up |
| Rnase2a | 10.97895997 | 0.000117107 | up |
| Cxcl2 | 10.16466796 | 0.000597527 | up |
| Saa3 | 9.881412082 | 0.009052154 | up |
| Cxcl3 | 9.854949016 | 0.000302282 | up |
| Orm1 | 9.833545928 | 0.000546581 | up |
| Stfa3 | 9.537557719 | 0.001435264 | up |
| Serpina3m | 9.514000351 | 1.40E-05 | up |
| Gm3776 | 9.345784172 | 0.000273684 | up |
| Gpr84 | 9.18558896 | 0.000276556 | up |
| Trim16 | 8.98679261 | 0.002090229 | up |
| Fkbp5 | 8.936537393 | 0.035743118 | up |
| Lcn2 | 8.878287918 | 0.001000406 | up |
| Mt2 | 8.568701623 | 0.003200637 | up |
| Saa1 | 8.484860619 | 0.000113699 | up |
| Tbc1d14 | -12.07147242 | 0.047689814 | down |
| Faim2 | -11.43818154 | 0.008686856 | down |
| Plekha6 | -10.78106268 | 0.007274948 | down |
| Ap3m2 | -10.62983796 | 0.000179915 | down |
| Snx14 | -9.763538779 | 4.84E-05 | down |
| Cobl | -9.185933427 | 0.000416295 | down |
| Reps2 | -9.168038592 | 0.006706371 | down |
| Scn3a | -8.879354064 | 0.001763602 | down |
| Colq | -8.741692689 | 0.003498004 | down |
| Abca8a | -8.679559519 | 9.86E-05 | down |
| Fat3 | -8.556047713 | 0.000815426 | down |
| Ces1e | -8.394838755 | 0.000146471 | down |
| Cadps2 | -8.284942415 | 0.000161866 | down |
| Slc4a5 | -8.120380029 | 1.43E-05 | down |
| Map3k12 | -7.631634751 | 0.024675966 | down |
| Vsnl1 | -7.531950196 | 0.002016081 | down |
| Abcc9 | -7.471802968 | 0.003290819 | down |
| Scgb3a2 | -7.45636159 | 0.020707624 | down |
| Pmp22 | -7.207502455 | 0.021120106 | down |
| Stmn2 | -6.828019716 | 0.001063803 | down |

**Supplementary Table 3:** The specific description of the primer sequences

| Name | Forward primer | Reverse primer |
| --- | --- | --- |
| Nkx2-1 | 5’-ACTGCTGGACGACTTCTTCTTCTTC -3’ | 5’-GGAGGAGGAGGAGGAGGAGGAG-3’ |
| Tbx2 | 5’-ACTGGTGTGGAGGATGCTGGAG -3’ | 5’-GGAAGACCAAGCGGACATGACTC-3’ |
| Atf5 | 5’-TCTCCGCTCACACCGTCTCTTC- 3’ | 5’-GAAGACAGGCACCAAGGCGAAG- 3’ |
| mmu-miR-135b-3p | 5’-CGCGATGTAGGGCTAAAAGC  -3’ | 5’-AGTGCAGGGTCCGAGGTATT  -3’ |
| RT Primer | 5’-GTCGTATCCAGTGCAGGGTCCGAGGTATTCGCACTGGATACGACCCCATG-3’ |  |
| mmu-miR-7062-5p | 5’-TGGAGGCCAGCTTGTGGA -3’ | 5’-AGTGCAGGGTCCGAGGTATT  - 3’ |
| RT Primer | 5’-GTCGTATCCAGTGCAGGGTCCGAGGTATTCGCACTGGATACGACAGACAC-3’ |  |
| mmu-miR-671-5p | 5’-AGGAAGCCCTGGAGGGG- 3’ | 5’-AGTGCAGGGTCCGAGGTATT- 3’ |
| RT Primer | 5’-GTCGTATCCAGTGCAGGGTCCGAGGTATTCGCACTGGATACGACCTCCAG-3’ |  |
| LNC_000068 | 5’-GGAAGGACACAGAACCACAGAAGG-3’ | 5’-CGGCAGAATGACAGAGGAGAAAGG -3’ |
| RP24-127D13.2 | 5’-CCAGTGCCAACAGAATACCCAGTC-3’ | 5’-CCAAGCCCCTTAGCCAGCAAAG-3’ |
| RP24-150D8.2 | 5’-GAGAAAGCAACCACCACGGGAAG- 3’ | 5’-AAGGTGCCAGGCGTGTATGTTG  -3’ |
| RP23-46P4.7 | 5’-TGCGGCGGGAGACTTTAGGTAG- 3’ | 5’-GGTTAAGGCGTCGGATGGCTTC- 3’ |
| GAPDH | 5’-AAATGGTGAAGGTCGGTGTGAAC-3’ | 5’-CAACAATCTCCACTTTGCCACTG-3’ |
| U6 | U6-S  5’-CTCGCTTCGGCAGCACA-3’ | U6-A  5’-AACGCTTCACGAATTTGCGT-3’ |

**Supplementary Table 4:** The top 10 enriched GO terms of biological process (BP), cellular component (CC), and molecular function (MF) are shown

| Category | Description | pvalue | GeneRatio | Count |
| --- | --- | --- | --- | --- |
| BP | response to wounding | 4.36E-07 | 23/720 | 23 |
| BP | multicellular organismal development | 1.76E-06 | 76/720 | 76 |
| BP | single-organism developmental process | 2.56E-06 | 86/720 | 86 |
| BP | inflammatory response | 2.65E-06 | 20/720 | 20 |
| BP | positive regulation of biological process | 2.84E-06 | 85/720 | 85 |
| BP | positive regulation of cellular process | 2.85E-06 | 78/720 | 78 |
| BP | cellular response to chemical stimulus | 3.27E-06 | 48/720 | 48 |
| BP | regulation of multicellular organismal process | 3.64E-06 | 51/720 | 51 |
| BP | regulation of developmental process | 4.14E-06 | 47/720 | 47 |
| BP | developmental process | 4.32E-06 | 86/720 | 86 |
| CC | cell junction | 3.33E-05 | 24/720 | 24 |
| CC | extracellular matrix | 5.00E-05 | 11/720 | 11 |
| CC | cytoplasm | 0.00010653 | 125/720 | 125 |
| CC | extracellular matrix component | 0.00012728 | 7/720 | 7 |
| CC | extracellular region part | 0.00018663 | 33/720 | 33 |
| CC | proteinaceous extracellular matrix | 0.00020225 | 7/720 | 7 |
| CC | basement membrane | 0.00026883 | 6/720 | 6 |
| CC | plasma membrane part | 0.00048932 | 41/720 | 41 |
| CC | senescence-associated heterochromatin focus | 0.00055281 | 2/720 | 2 |
| CC | extracellular space | 0.0014022 | 28/720 | 28 |
| MF | protein binding | 3.42E-06 | 111/720 | 111 |
| MF | receptor binding | 0.00011614 | 32/720 | 32 |
| MF | identical protein binding | 0.00020114 | 33/720 | 33 |
| MF | growth factor binding | 0.00027789 | 7/720 | 7 |
| MF | transmembrane receptor protein tyrosine phosphatase activity | 0.0005149 | 2/720 | 2 |
| MF | transmembrane receptor protein phosphatase activity | 0.0005149 | 2/720 | 2 |
| MF | transcriptional activator activity, RNA polymerase II core promoter proximal region sequence-specific binding | 0.0015683 | 9/720 | 9 |
| MF | 5'-deoxyribose-5-phosphate lyase activity | 0.0017551 | 2/720 | 2 |
| MF | cell adhesion molecule binding | 0.0021975 | 7/720 | 7 |
| MF | transcription factor activity, RNA polymerase II core promoter proximal region sequence-specific binding | 0.0022652 | 11/720 | 11 |

**Supplementary Table 5:** The top 20 KEGG enrichment analysis

| pathway_term | rich_factor | P-Value | gene_number |
| --- | --- | --- | --- |
| NF-kappa B signaling pathway | 0.06 | 0.000703275 | 6 |
| ECM-receptor interaction | 0.056818182 | 0.002458577 | 5 |
| Focal adhesion | 0.033816425 | 0.005965364 | 7 |
| Ubiquinone and other terpenoid-quinone biosynthesis | 0.2 | 0.006170481 | 2 |
| PI3K-Akt signaling pathway | 0.025641026 | 0.010763064 | 9 |
| Leishmaniasis | 0.046153846 | 0.030880265 | 3 |
| Amoebiasis | 0.033613445 | 0.035391361 | 4 |
| B cell receptor signaling pathway | 0.04109589 | 0.040894183 | 3 |
| Adherens junction | 0.040540541 | 0.04224774 | 3 |
| Galactose metabolism | 0.060606061 | 0.047868691 | 2 |
| Small cell lung cancer | 0.035294118 | 0.058582174 | 3 |
| Malaria | 0.041666667 | 0.089498189 | 2 |
| Notch signaling pathway | 0.040816327 | 0.092558789 | 2 |
| Amyotrophic lateral sclerosis (ALS) | 0.038461538 | 0.101917794 | 2 |
| TNF signaling pathway | 0.027522936 | 0.102656262 | 3 |
| Glutathione metabolism | 0.037735849 | 0.105093564 | 2 |
| Toxoplasmosis | 0.026548673 | 0.11099712 | 3 |
| Purine metabolism | 0.022346369 | 0.11233256 | 4 |
| Legionellosis | 0.034482759 | 0.121354786 | 2 |
| Inositol phosphate metabolism | 0.032786885 | 0.131388214 | 2 |

**Supplementary Table 6:** The five main steps are as follows

Step1: Exon number screening of transcripts: a large number of transcripts with low expression and low confidence were filtered from the splicing results of transcripts, and transcripts with exon number ≥ 2 were selected.

Step2: Transcript length screening: select transcripts with transcript length > 200bp.

Step3: Transcription known annotation screening: Cuffcompare software was used to screen out transcripts that overlapped with exon region of database annotation, and lncRNAs that overlapped with exon region of this spliced transcript in database were included in subsequent analysis as database annotation lncRNAs.

Step4: Transcription expression screening: Cuffquant was used to calculate the expression level of each transcript, and the transcript with FPKM ≥ 0.5 was selected.

Step5: Coding potential screening: whether the transcript has coding potential is the key condition to determine whether the transcript is lncRNA.

**Supplementary Table 7:** The details of calculating the coding potential using the four tools

1. CNCI (Coding-Non-Coding-Index) (v2) profiles adjoining nucleotide triplets to effectively distinguish protein-coding and non-coding sequences independent of known annotations. We use CNCI with default parameters.

2. CPC (Coding Potential Calculator) (0.9-r2) mainly through assess the extent and quality of the ORF in a transcript and search the sequences with known protein sequence database to clarify the coding and non-coding transcripts. We used the NCBI eukaryotes' protein database and set the e-value ‘1e-10’in our analysis.

3. Pfam-sca: We translated each transcript in all three possible frames and used Pfam Scan (v1.3) to identify occurrence of any of the known protein family domains documented in the Pfam database (release 27; used both Pfam A and Pfam B). Any transcript with a Pfam hit would be excluded in following steps. Pfam searches use default parameters of -E 0.001 --domE 0.001.

4. PhyloCSF (phylogenetic codon substitution frequency) (v20121028) examines evolutionary signatures characteristic to alignments of conserved coding regions, such as the high frequencies of synonymous codon substitutions and conservative amino acid substitutions, and the low frequencies of other missense and non-sense substitutions to distinguish protein-coding and non-coding transcripts. We build multi-species genome sequence alignments and run phyloCSF with default parameters.
